# Comprehensive knowledge and uptake of cervical cancer screening is low among women living with HIV/AIDS in Northwest Ethiopia

**DOI:** 10.1186/s40661-017-0057-6

**Published:** 2017-12-19

**Authors:** Daniel Asfaw Erku, Adeladlew Kassie Netere, Amanual Getnet Mersha, Sileshi Ayele Abebe, Abebe Basazn Mekuria, Sewunet Admasu Belachew

**Affiliations:** 1 Department of Clinical Pharmacy, School of Pharmacy, College of Medicine and Health Sciences, University of Gondar, Chechela Street, Lideta Sub city Kebele 16, P.O. Box: 196, Gondar, Ethiopia; 2 Department of Gynecology and obstetrics, College of Medicine and Health Sciences, University of Gondar, Chechela Street, Lideta Sub city Kebele 16, Gondar, Ethiopia; 3 Department of Pharmacology, School of Pharmacy, University of Gondar, Chechela Street, Lideta Sub city Kebele 16, Gondar, Ethiopia

**Keywords:** Cervical cancer, Ethiopia, HIV/AIDS, Screening, Knowledge, Women

## Abstract

**Background:**

In Ethiopia, cervical cancer is ranked as the second most common type of cancer in women and it is about 8 times more common in HIV infected women. However, data on knowledge of HIV infected women regarding cervical cancer and acceptability of screening is scarce in Ethiopia. Hence, the present study was aimed at assessing the level of knowledge of about cervical cancer and uptake of screening among HIV infected women in Gondar, northwest Ethiopia.

**Methods:**

A cross sectional, questionnaire based survey was conducted on 302 HIV infected women attending the outpatient clinic of University of Gondar referral and teaching hospital from March 1 to 30, 2017. Descriptive statistics, univariate and multivariate logistic regression analysis were also performed to examine factors associated with uptake of cervical cancer screening service.

**Results:**

Overall, only 64 (21.2%) of respondent were knowledgeable about cervical cancer and screening and only 71 (23.5%) of respondents were ever screened in their life time. Age between 21 and 29 years old (AOR = 2.78, 95% CI = 1.71–7.29), perceived susceptibility to develop cervical cancer (AOR =2.85, 95% CI = 1.89–6.16) and comprehensive knowledge of cervical cancer (AOR = 3.02, 95% CI = 2.31–7.15) were found to be strong predictors of cervical cancer screening service uptake.

**Conclusion:**

The knowledge and uptake of cervical cancer screening among HIV infected women was found to be very poor. Taking into consideration the heightened importance of comprehensive knowledge in boosting up the number of participants towards cervical cancer screening services, different stakeholders working on cancer and HIV/AIDS should provide a customized health promotion intervention and awareness creation to HIV-infected women, along with improving accessibility of cervical cancer screening services in rural areas.

## Background

Cancer of the cervix, mainly attributed to persistent infection with a high risk oncogenic Human papillomavirus (HR-HPV), is one of the most common type of women’s cancer globally, with more than 90% of new cases occurring in developing and resource-limited countries [1–3]. It is also associated with a higher rate of mortality with over 150,000 global mortality reported only in 2012, of which 87% of death occurred in developing countries [1, 2]. In Ethiopia, cervical cancer is ranked as the second most common type of cancer in women with crude incidence rate of 16.3 per 100,000 populations annually. In 2012 only, more than 27 million women of reproductive age in Ethiopia were at risk of developing cervical cancer and jeopardized the lives of more than 4500 women [4].

Evidences showed that, immunosuppression and low CD4 counts caused by HIV infection predisposes women living with HIV infection at an increased risk for cervical cancer and the development of squamous intraepithelial lesions [5–9]. Cervical cancer is about 8 times more common in HIV infected women than none infected ones [5]. From around 35 new cases diagnosed annually per 100, 000 population in sub-Saharan African countries, about 60% of the cases diagnosed among patients living with HIV infection [10, 11]. In a study done in southern Ethiopia, around 22% women infected with HIV were positive for precancerous cervical cancer [8].

Despite its preventable cause, global cervical cancer incidence rate is expected to be doubled by 2025 [12]. While performing regular screening is known to prevent the disease by a significant percentage [13, 14], the acceptability of regular screening in Ethiopia is limited and covers less than 1% of women [12, 15]. Moreover, comprehensive data on knowledge of women living with HIV/AIDS regarding cervical cancer and acceptability of screening is lacking in Ethiopia, which limits the development of cancer prevention efforts in these patient populations. Hence, the present study was aimed at assessing the level of knowledge about cervical cancer and uptake of screening among women living with HIV/AIDS in Gondar, northwest Ethiopia.

## Methods

### Study design and setting

A hospital-based cross-sectional survey was employed on 302 women attending ART clinic at University of Gondar Referral and Teaching Hospital (UoGRTH), northwest Ethiopia. UoGRTH is located in Gondar town, northwest Ethiopia, 738 km away from Addis Ababa (the capital city of Ethiopia).

### Sampling and recruitment strategies

All HIV infected women above age of 17 years who visited the outpatient clinic of UoGRTH for follow up and medication refill were taken as a study population. Single population proportion formula was used with the assumption of 95% confidence interval, 5% margin of error, the proportion (p) of cervical cancer screening in women living with HIV/AIDS (11%) [16] and 5% for possible non-response was taken to determine a final sample size of 317. A systematic random sampling technique was then applied to select participants until the final sample size was attained.

### Survey instrument

Data collection was performed by two of the principal investigators through interviewer-administered questionnaire. The investigators were properly trained on the instrument and ways of approaching the patients and securing potential participants permission for the interview prior to the commencement of the study. The data collection tool was developed after a thorough literature review of the published studies [17–20] and was primarily prepared in English. This was translated to local language (Amharic) and then back to English by an expert in the area in order to ensure that the translated version gives the proper meaning. The data collection instrument was also pretested on 20 women who were not included in the final analysis and relevant modifications were instituted before the commencement of actual data collection. The final questionnaire divided into three main parts. The first section was focusing on the socio-demographic and disease related information including age, marital status, educational level, CD4 count and WHO clinical stage. The second section, having 30 yes/no or true/false questions, assessed the knowledge about cervical cancer (CC) screening with five subcategories (risk factors for CC, prevention of CC, clinical symptoms of CC, benefits of screening, and meaning of positive results). The third section asked respondents regarding the uptake of cervical cancer screening services. Total scores for each category were then summed up to determine an overall score with a maximum score of 30. Using published literature as a reference [17], we classify respondents with a score of 60% or more as knowledgeable. The number of women who achieved this for any one score is defined as the knowledge rate. The third section included questions about the respondents’ uptake of CC screening.

### Statistical analysis

All the statistical analyses were done using Statistical Package for the Social Sciences (SPSS) software version 21.0 for Windows (SPSS Inc., Chicago, IL). Frequencies and percentages were used to express different variables. Univariate and multivariate logistic regression analysis were used to determine predictors of knowledge about cervical cancer and acceptability of screening. The results were adjusted for patients’ demographic and disease characteristics. Odds ratio (OR) with 95% CI were computed along with corresponding *p*-value (*p* < 0.05) as cut off points for determining statistical significance.

### Ethical considerations

This study was approved by the ethical review committee of University of Gondar with an approval number of UoG-SoP-92/2017. Written informed consent from the respondents was also obtained before conducting this study. Participants’ information obtained was kept confidential.

### Operational definitions

CD4 Count: It is a laboratory value that measures the number of CD4 T lymphocytes (CD4 cells) in a sample of blood. In people with HIV, the CD4 count is the most important laboratory indicator of immune function and the predictor of HIV progression. Healthy individuals CD4 count ranges from 500 to 1700 cells/mm^3^. However, in HIV positive individuals the CD4 count sharply drops down to less than 500 cells/mm^3^, which shows immunodefiency.

WHO clinical Stage: WHO clinical staging is based on clinical findings that guide the diagnosis, evaluation, and management of HIV/AIDS, and it does not require a CD4 cell count. This staging system is used in many countries to determine eligibility for antiretroviral therapy, particularly in settings in which CD4 testing is not available like the case of Ethiopia. These stages are defined by specific clinical conditions or symptoms. With this, clinical stages are categorized as 1 through 4, progressing from primary HIV infection to advanced HIV/AIDS [21].

## Results

### Characteristics of the study participants

Out of 317 patients approached, 302 of them were included in the study giving a response rate of 95.3%. The mean age of respondents was 33.72 years with a standard deviation of ±9.72. Majority of the respondents were urban residents (69.9%). A substantial proportion of respondents (36.7%) were at the stage of WHO clinical stage 2 and had a CD4 count of ≤500 cells/ul (66.9%). The sociodemographic and disease characteristics of study participants are depicted in Table 1.

**Table 1:** Sociodemographic characteristics and factors associated with uptake of cervical cancer screening, Gondar, 2017

Variables	Total	Screening		AOR (95% CI)
		No (*n* = 231)	Yes (*n* = 71)	
Age group, in years				
< 29	128 (42.4%)	111	17	1
30–39	105 (34.8%)	69	36	2.78 (1.71–7.29)
> 40	69 (22.8%)	51	18	2.61 (1 .89–5.17)
Residence				
Rural	91 (30.1%)	71	20	–
Urban	211 (69.9%)	160	51	–
Marital status				
Unmarried	123 (40.7%)	112	11	–
Ever married	179 (59.3%)	119	60	–
Educational status				
Illiterate	33 (10.9%)	28	5	1
Primary	145 (48%)	133	12	0.87 (0.33–1.79)
Secondary	80 (26.5%)	66	14	1.08 (0.54–1.91)
Tertiary	44 (14.6%)	4	40	0.41 (0.21–1.29)
Average monthly income				
< 100	148 (49%)	119	29	–
100–150	85 (28.2%)	59	16	–
> 150	69 (22.8%)	43	26	–
Age at first sex				
≤ 16	63 (20.9%)	46	17	–
> 16	239 (79.1%)	185	54	–
Had multiple sexual partner				
No	123 (40.7%)	89	34	1
Yes	179 (59.3%)	142	37	1.01 (0.43–1.72)
Comprehensive knowledge about CC				
Not knowledgeable	238 (78.8%)	129	9	1
Knowledgeable	64 (21.2%)	2	62	3.02 (2.31–7.15)
CD4 count				
< 500 cells/ul	202 (66.9%)	170	32	–
> 500 cells/ul	100 (33.1%)	61	39	–
WHO clinical stage				
One	91 (30.1%)	80	11	1
Two	111 (36.7%)	90	21	0.62(0.39–1.72)
Three	67 (22.2%)	52	15	1.01(0.41–1.52)
Four	33 (11%)	9	24	0.91(0.40–1.69)
Perceived susceptibility				
None receptive	118 (39.1%)	105	13	1
Receptive	184 (60.9%)	126	58	2.85 (1.89–6.16)

### Knowledge and uptake of cervical cancer screening

The majority of respondents in this study 265 (87.7%) had heard about cervical cancer and its screening. The average total knowledge score was found to be 10.8 ± 5.20 (a range possible from 0 to 30), with a mean score of 1.64 ± 1.27 for the risk factors of CC (a range possible from 0 to 7), 1.77 ± 1.81 for preventive measures of CC (a range possible from 0 to 7), 2.74 ± 1.67 for clinical symptoms of CC (a range possible from 0 to 9), 1.71 ± 1.23 for benefits of screening (a range possible from 0 to 3) and 2.72 ± 1.07 for understanding about the positive results of CC (a range possible from 0 to 4). Overall, only 64 (21.2%) of respondent were knowledgeable about CC and screening as per the definition set in our study.

The majority of respondents correctly answered that CC is both preventable 238 (78.8%) and curable 223 (73.8%) disease. However, a significant proportion of respondents 103 (34.1%) didn’t know the risk factors of CC or identified only one 57 (18.9%) or two 45 (14.9%) risk factors. While a highest proportion of patients correctly identified “early onset of sexual activity” 112 (37.1%) as a risk factor for CC, hormonal contraceptive use and HPV infection were identified as risk factors for CC only by 57 (18.9%) and 53 (17.5%) of respondents respectively. Similarly, over half of the respondents 172 (56.9%) knew that CC screening could prevent CC occurrence. Yet, only 88 (29.1%) respondents believed that CC screening could enable early diagnosis of the disease. Even though majority of respondents knew at least one of the clinical symptoms of CC 274 (90.7%), a substantial proportion of respondents 63 (20.8%) incorrectly stated “vulvar itching or burning sensation” as one of the clinical symptoms of CC. The detailed frequency of correct answer for each knowledge items are presented in Table 2. According to the findings our study, only 71 (23.5%) of respondents were ever tested for CC in their life time, of which 29 (40.8%) of them screened after 1 year of HIV/AIDS diagnosis. Among the 231 (76.5%) of respondents who were not screened for CC, absence of symptoms 205 (88.7%) and emotional barriers like fear of test result 164 (71%) and embarrassment 159 (68.8%) were the main reasons for not undergoing CC screening (Table 3).

**Table 2:** Frequency of correct answer for knowledge items about CC among participants, Gondar, Ethiopia, 2017

Knowledge items	Correct answers (%)
Risk factor for CC	
Prolonged use of oral contraceptive	57 (18.9%)
Sexually transmitted infection	77 (25.5%)
Early onset of sexual activity	112 (37.1%)
Smoking	54 (17.9%)
Multiple sexual partner	49 (16.2%)
History of HPV infection	53 (17.5%)
Aged 30–65	65 (21.5%)
Symptoms of cervical cancer	
Bleeding and pain after sexual intercourse	60 (19.9%)
Vulvar itching or burning sensation	63 (20.8%)
Post-menopausal bleeding	54 (17.9%)
Excessive vaginal discharge	71 (23.5%)
Abnormal vaginal discharge	68 (22.5%)
Inter-menstrual bleeding	67 (22.2%)
Longer or heavier menstrual periods	55 (18.2%)
Pelvic pain	48 (15.9%)
Urinary frequency, urgency	38 (12.6%)
Preventive measures for CC	
CC screening	172 (56.9%)
Reduce numbers of sexual partners	61 (20.2%)
Vaccine for HPV	27 (8.9%)
Late marriage and late childbirth	21 (6.9%)
No smoking	57 (18.9%)
Consistent condom use	29 (9.6%)
Prompt treatment of STIs	71 (23.5%)
Benefits of screening for CC	
Early detection	71 (23.5%)
Early diagnosis	88 (29.1%)
Early treatment	101 (33.4%)
Understanding of the positive results	
Negative screening result means cervix without any lesion, needing no more screening	142 (47%)
Positive screening result means suffering from CC	224 (74.2%)
Positive screening result means there is cervical lesion, it needs further diagnosis	76 (25.2%)
CC is a curable disease	223 (73.8%)

**Table 3:** Acceptance of CC screening service among study participants, Gondar, Ethiopia, 2017

Variables	Frequency (%)
Have you ever had CC screening in your life time?	
No	231 (76.5%)
Yes	71 (23.5%)
If yes, when was the last time you screened for cervical cancer? (*N* = 71)	
Before HIV/AIDS diagnosis	19 (26.8%)
Within 1 year of HIV/AIDS diagnosis	23 (32.4%)
After 1 year of HIV/AIDS diagnosis	29 (40.8%)
If no, what are the reasons for not being screened? (*N* = 231)	
Absence of symptoms	205 (88.7%)
High cost of the test	64 (27.7%)
Not prescribed by the doctor	76 (32.9%)
Embarrassment	159 (68.8%)
Time consuming	44 (19%)
Fear of test result	164 (71%)
Screening center too far	87 (37.7%)
No reason	46 (19.9%)
Others^a^	19 98.2%)
Are you willing to be screened in the near future? (*N* = 302)	
No	88 (29%)
Yes	214 (71%)

^a^Others include Religious denial, partner acceptance, no symptom

### Predictors of CC screening service uptake

Logistic regression analysis was employed to assess possible associations between different sociodemographic variables and women’s CC screening service uptake. According to the results from bivariate logistic regression, there were statistically significant differences in age, history of multiple sexual partners, educational status, WHO clinical stage, comprehensive knowledge of CC and screening and perceived susceptibility to develop CC between women who underwent CC screening and those who didn’t. Variables that were significantly associated with CC screening service uptake in the bivariate analysis (those with *p*-value < 0.20) were further examined in multivariate logistic regression. Accordingly, age, perceived susceptibility to develop CC and comprehensive knowledge of CC and screening remained to be significant in the multivariate logistic model. The odds of CC screening service uptake among women in the age range of 30–39 years were 1.78 times higher than women aged less than 29 years old (AOR = 2.78, 95% CI = 1.71–7.29). The odds of CC screening uptake among women who had positive perception on their susceptibility to develop CC were 1.85 times higher than those who had negative perception (AOR =2.85, 95% CI = 1.89–6.16). Similarly, the odds of undergoing CC screening among women who had a comprehensive knowledge on CC and screening were 2.02 times higher than those who didn’t have comprehensive knowledge on CC and screening (AOR = 3.02, 95% CI = 2.31–7.15).

## Discussion

Exploring the comprehensive knowledge towards the causative/risk factors, benefits of screening, pertinent manifestations and prevention of cervical cancer is so indispensable in women care. According to the finding of this study, majority of (87.7%) respondents heard about cervical cancer and its screening. The overall knowledge rate of CC was 21.2%, which is higher compared to the study done in Nigeria where 11.8% of the rural and 17.6% of urban women had knowledge of CC [19]. In line with this, the average total knowledge score women had was found to be merely 10.8 ± 5, which is higher than the study conducted in china, which is 6.91 ± 3.42 [18]. The difference in knowledge score could be attributed to the difference in time period and the characteristics of the study population as both of the studies were conducted among the general population in Nigeria and China and did not account whether the respondents had HIV/AIDS or not. This could potentially account for the variation in the level of knowledge as patients living with HIV/AIDS expected to have a higher level of awareness about CC due to their frequent contact with healthcare providers compared to the general population. In our study, only 33.8% of the study respondents were capable of identifying one or two risk factors for CC, which is lower than the study conducted among rural communities of South Africa in which about 64% of the respondents gave one or more risk factors [22]. This might be due to the absence of a comprehensive cancer prevention and treatment center in Ethiopia unlike countries like South Africa..Majority of respondents in this study (73.8%) believed that CC is curable, which is lower than the study conducted in China (80.8%) [18]. This might be due to the difference in the background of study population as the study conducted in China included respondents from Wufeng, a high-incidence region of cervical cancer in China.

According to the findings of our study, only 23.5% of respondents were ever tested for CC in their life time, which is significantly higher compared with the study done in among patients living with HIV/AIDS in Addis Ababa, Ethiopia, where only 11.5% of women screened for CC [16]. The uptake of screening in our study is also higher compared with the study conducted in Nigeria (9.4%) [23]. The enhanced uptake of screening service in our study could be partially explained by the increased nation-wide advocacy, community sensitization and awareness creation about the CC screening that has been put into effect in recent years. It might also be due to the improved expansion and access of screening centers across the country and integration of CC screening into the standard care for women who are living with HIV/AIDS. Yet, the proportion of women screened for CC in our study is still low compared to developed countries such as Ottawa (58%) [24], despite the recent effort to screen all HIV positive women who are on antiretroviral therapy (ART) who were not screened before. Among 76.5% of patients who were not screened in our study, absence of symptoms (88.7%) and emotional barriers like fear of test result (71%) and embarrassment (68.8%) were the main reasons for not undergoing screening, which was consistent with the study conducted in China [18]. According to the results from multivariate logistic regression analysis, age, perceived susceptibility to develop CC and comprehensive knowledge of CC and screening remained to be strong predictors of CC screening service uptake. The odds of CC screening service uptake among women in the age range of 30–39 years were 2.78 times higher than women aged 21–29 years old. Similar findings were also reported both in developing and developed countries [25, 26]. This is not surprising as women at the age of 30s and 60s are more likely to be symptomatic due to the bimodal distribution nature of the CC, which may enhance their probability of screening for CC. Similarly, the odds of CC screening uptake among women who had positive perception on their susceptibility to develop CC were 2.85 times higher than those who had negative perception, which could be explained by the assumption of behavioral model, which assumes that belief and attitudes, including self-vulnerability to illness, are important predictors of their health-related activities [27]. Furthermore, the odds of undergoing CC screening among women who had a comprehensive knowledge on CC and screening were 3.02 times higher than those who didn’t have comprehensive knowledge on CC and screening, which corroborates the findings of studies conducted among patients living with HIV/AIDS in Addis Ababa, Ethiopia and Botswana [16, 28].

### Limitation of the study

Even though this survey highlights an area of research where there is lack of literature in Ethiopia, caution should be exercised when generalizing to other regions in Ethiopia as the study was a cross-sectional and conducted only in Gondar, northwest Ethiopia. Nevertheless, this survey has significant implications for improving uptake of CC screening services and provide a foundation for planning future in-depth research prior to developing educational materials. A larger-scale and multi centered survey that includes more diverse participants is warranted to validate our findings and to provide more accurate findings. Furthermore, our study could be used as an input for future studies aiming at exploring the difference in knowledge about cervical cancer and cervical cancer screening uptake experience between HIV positive and negative women.

## Conclusion and recommendation

The results of the present study revealed that the knowledge and uptake of cervical cancer screening among HIV infected women was very poor. Our findings emphasize the need to reform the existing national strategies of cervical cancer screening so as to strengthen the health education and promotion, beyond providing screening services. Taking into consideration the heightened importance of comprehensive knowledge in participating in cervical cancer screening services, different stakeholders working on cancer and HIV/AIDS should provide a customized health promotion intervention and awareness creation among HIV-infected women. Furthermore, interventions should focus on overcoming the identified barriers for not being screened including improving accessibility of cervical cancer screening services in rural areas.

## Abbreviations

CC: Cervical cancer
CI: Confidence interval
HIV/AIDS: Human immunodeficiency virus/Acquired immune deficiency syndrome
HPV: Human papillomavirus
HR-HPV: High risk oncogenic Human papillomavirus
OR: Odds ratio
SPSS: Statistical package for the social sciences
UoGRTH: University of Gondar Referral and Teaching Hospital
WHO: World health organization
